# Supplementary material for: A deep learning framework for characterization of genotype data
Source: G3 (Bethesda). 2022 Jan 25;12(3):jkac020. doi: 10.1093/g3journal/jkac020 (PMC8896001; doi:10.1093/g3journal/jkac020)
Supplement: jkac020_Supplementary_Data [file jkac020_supplementary_data.pdf]

Supplementary Material

A deep learning framework for characterization of genotype data

Kristiina Ausmees, Carl Nettelblad

Optimization of model design and training of GCAE

The fundamental structure of the model was chosen based on empirical experience from previous experiments (not published) and related literature. This includes the types of layers used (convolutional, maxpooling, fully-connected, and upsampling by means of nearest-neighbor interpolation) and the ordering of these. We had also found benefits of the use of residual connections and dropout for the weights of fully-connected layers, except those on either side of the latent representation. Previous experience had also shown that the use of the two sets of marker-specific variables had improved model performance, and indicated suitable locations to insert them in the decoder. For demonstration, we show the the difference in F1 scores for the models and hyperparameters presented in the main text and in Table 3 with and without the use of marker-specific variables in Figure 1.

Based on this fundamental structure, an evaluation of different architecture settings, shown in Table 1, was performed. These tests were done using a limited set of hyperparameter combinations. Once the final model architecture and settings had been decided, a larger search among the hyperparameter values shown in Table 2 was performed. We note that not all combinations of settings in the two tables were evaluated for the different applications.

Several additional strategies for improving the training process were also evaluated throughout the above described process. We found that using a linear activation function on the outermost fully-connected layers led to a more stable training that was less likely to collapse and avoided artefacts in the latent representation. Data-augmentation by means of setting input genotypes to missing was found to reduce overfitting. This was implemented by randomly setting f genotypes to missing in each batch, where f was randomly selected from $[0.0, 0.1, 0.2, 0.3, 0.4]$. Introducing errors with a probability of 0.2 by setting all missing values in a batch to genotype values drawn from a uniform distribution was also found to improve performance.

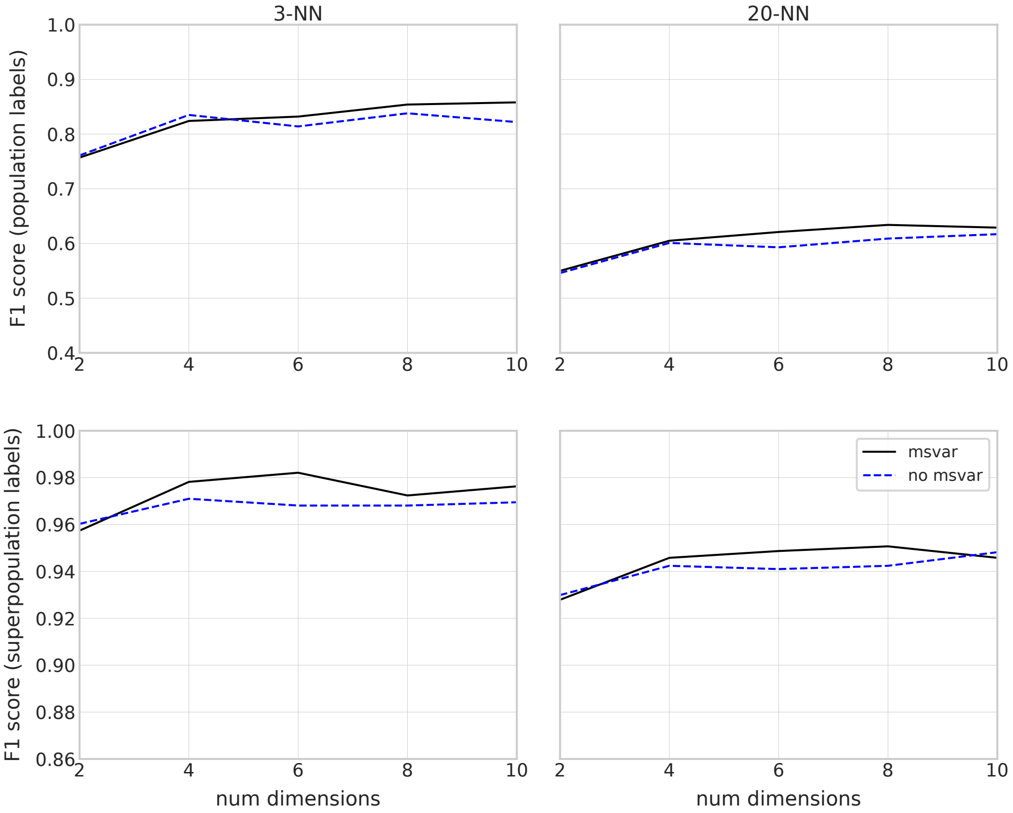

Figure 1: F1 scores for 3-NN and 20-NN classification models based on dimensionality reduction of GCAE with and without marker-specific variables using 2-10 dimensions. Top plots show results for using populations as labels in the classification, and bottom ones for using superpopulations as labels. The model architecture and hyperparameters used are the same as described in the main article text and in Table 3

Parameter	Values
kernel size convolution	3, 5, 10
pool size maxpool	2, 3, 5
stride maxpool	2, 3
number of filters/kernels convolution	8, 16, 32
dropout rate	0.0, 0.01, 0.1
number of units in fully-connected layers	15, 25, 50, 75

Table 1: Model architecture settings

Parameter	Values
learning rate	3.2e-06, 1e-05, 3.2e-05, 0.0001, 0.00032, 0.001, 0.0032, 0.01
exponential decay rate	0.92, 0.94, 0.96, 0.98
decay every epochs	10, 20
regularization factor	3.2e-09, 1e-08, 3.2e-08, 1e-07. 3.2e-07, 1e-06, 3.2e-06
noise std	10.0, 3.2, 1.0, 0.32, 0.1, 0.032, 0.01
batch size	30, 50, 60, 75, 100

Table 2: Hyperparameters evaluated for GCAE

Parameter	DR-2	DR-4	DR-6	DR-8	DR-10	GC	OA
kernel size convolution	5	5	5	5	5	5	10
number of filters/kernels convolution	8	8	8	8	8	8	8
pool size maxpool	5	5	5	5	5	5	5
stride maxpool	2	2	2	2	2	2	2
dropout rate	0.01	0.01	0.01	0.01	0.01	0.01	0.01
number of units in fully-connected layers	75	75	75	75	75	75	75
learning rate	0.01	0.00032	0.00032	1.00e-05	1.00e-05	0.001	0.0001
exponential decay rate	0.92	0.92	0.92	0.98	0.98	0.94	0.98
decay every epochs	10	10	10	20	20	10	20
regularization factor	1e-08	1e-06	1e-06	1e-07	3.2e-08	1e-08	3.2e-09
noise std	0.032	0.32	1.0	3.2	10	0.032	3.2
batch size	60	60	60	30	30	60	30

Table 3: Final architecture settings and hyperparameters used for the dimensionality reduction (DR), genetic clustering (GC) and output analysis (OA) models.

Final implementations

Table 3 shows the architecture settings and hyperparameters used to obtain the presented dimensionality reduction (DR) and genetic clustering (GC) results, as well as the model selected for analysis of output genotypes (OA). For the DR results with more than 2 dimensions, the best model was selected based on evaluating the F1 score of a 3-NN population classification model. For GC and 2-dimensional DR, selection was done based on the F1 score, genotype concordance of the reconstructed data as well as inspection of the visualization. For OA, the model that yielded the highest number of non-fixed sites for LD analysis was selected. In all cases, the epoch at which to stop training was selected as the one where the validation loss was minimized, using a patience of 300 epochs.

Hyperparameter tuning of popvae

A grid search of depth, width and batch size was performed for popvae, values are shown in Table 4. The depth and width values evaluated are those that the built-in grid search option of popvae comprises. The values selected, for which results are shown in the main text, are shown for each number of dimensions in Table 5.

parameter	values
depth	3, 6, 10, 20
width	32, 64, 128, 256, 512
batch size	20, 32, 40

Table 4: Hyperparameters evaluated for popvae.

number of dimensions	depth	width	batch size
2	3	32	32
4	3	128	40
6	6	32	32
8	3	32	32
10	3	64	40

Table 5: Optimal hyperparameters for popvae for different number of dimensions, selected based on highest F1 score for a 3-NN population classification model.

Hyperparameter tuning of t-SNE

A grid search of learning rate and perplexity was performed for t-SNE, values are shown in Table 6. Default values were used for all other parameters and settings. The values selected, for which results are shown in the main text, are shown for each number of dimensions in Table 7.

parameter	values
learning rate	43, 170, 200, 225, 250, 300, 350, 400, 450, 500, 650, 600
perplexity	5, 10, 20, 30, 45, 50, 75,100, 125, 175, 200

Table 6: Hyperparameters evaluated for t-SNE.

number of dimensions	learning rate	perplexity
2	300	10
4	450	175
6	43	20
8	650	45
10	43	50

Table 7: Optimal hyperparameters for t-SNE for different number of dimensions, selected based on highest F1 score for a 3-NN population classification model.

Hyperparameter tuning of UMAP

A grid search of the hyperparameters number of neighbors, minimum distance, and spread was performed for UMAP for 2,4,6,8 and 10 number of dimensions. The values considered are shown in Table 8. Default values were used for all other parameters and settings. Optimal hyperparameters were selected based on the most accurate resulting 3-NN population classification model, measured using the F1-score. Table 9 shows the selected hyperparameters for UMAP for the different numbers of dimensions.

parameter	values
number of neighbors	3, 5, 10, 20, 50, 100, 200
minimum distance	0.05, 0.1, 0.25, 0.5, 0.75, 1.0
spread *	0.05, 0.1, 0.25, 0.5, 0.75, 1.0

Table 8: Hyperparameters evaluated for UMAP.
*The implementation used requires spread to be larger than or equal to minimum distance, so only such combinations were considered.

dimensions	num neighbors	min distance	spread
2	3	0.05	0.25
4	3	0.05	0.25
6	3	0.1	0.1
8	3	0.05	0.05
10	3	0.1	0.1

Table 9: Optimal hyperparameters for UMAP for different number of dimensions, selected based on highest F1 score for a 3-NN population classification model.

In [1], number of neighbors is described as influencing the tradeoff between fine-grained and large-scale manifold features, with smaller values resulting in detailed structure being captured at a loss of "big picture" information, and larger values keeping more large-scale manifold structures. Minimum distance is explained as affecting how closely points end up to each other in the output, where low values result in densely packed points, with manifold structure more likely to be accurately represented. In the code documentation of the implementation used, spread is described as the effective scale of the points, which in combination with minimum distance determines the degree of aggregation of points into clusters.

Thus, there is a tradeoff between local and global behavior for UMAP that can be controlled using the hyperparameters. Our optimization criteria favor local behavior, which is visible in the results in the main text. UMAP outperformed the other methods for the population classification model, but not for the superpopulation classification model, with particularly poor comparative performance for the 20-NN model.

In order to get a more comprehensive view of the behavior of UMAP, we also evaluate its performance for different hyperparameter values. For this, we consider reduction to 2 dimensions, and select hyperparameter values that cover the ranges considered for number of neighbors, minimum distance and spread. Table 10 shows the model identifiers and hyperparameter combinations considered. Also shown are two metrics of classification performance: F1 scores for a 3-NN population classification model and a 20-NN superpopulation classification model, reflecting local and global clustering performance. Also shown in the table are the corresponding metrics for the GCAE, PCA and t-SNE models for 2 dimensions from the main article text. Figures 2 - 4 show the dimensionality reduction results for the UMAP models.

It is visible that the hyperparameters have a large effect on the resulting dimensionality reduction. Increasing the number of neighbors decreases the tendency towards small, isolated clusters, and lower minimum distance and spread give more densely packed points. The F1 score of the 3-NN population classification model tends to be higher for lower neighbor counts, which is expected as those values promote local structures being captured. The more global performance, as measured by F1 score for a 20-NN superpopulation classification model, has less of a clear trend and seems to be less affected by the hyperparameter values. All models considered in Table 10 had lower F1 scores for this metric than GCAE, t-SNE and PCA. The results thus confirm the tradeoff between local and global performance for UMAP, whereas we found that for GCAE, classification of superpopulations tended to improve with that of populations, suggesting at a qualitative difference between the two methods.

We also note that the UMAP projections differ from those of PCA and GCAE in terms of the relative positions of the superpopulations. For the latter two, there is a tendency towards a gradient with Africa and East Asia at opposite ends, with Middle Eastern, European and South/Central Asian populations between them, roughly in this order. This pattern is evident in the PCA plot, and we also noted that GCAE results consistently showed the same tendency

when performing the hyperparameter optimization. In the UMAP plots, the African and East Asian clusters tend to appear closer together, often with the European cluster at the extreme end of the larger collection of clusters. This suggests systematic differences in the nature of the global patterns captured by the dimensionality reduction between UMAP and the other methods.

model id	num neighbors	min distance	spread	F1 score 3-NN (population labels)	F1 score 20-NN (superpopulation labels)
1	3	0.05	0.05	0.847	0.825
2	3	0.05	0.5	0.853	0.798
3	3	0.05	1.0	0.786	0.828
4	3	0.5	0.5	0.814	0.857
5	3	0.5	1.0	0.719	0.801
6	3	1.0	1.0	0.71	0.827
7	20	0.05	0.05	0.6	0.777
8	20	0.05	0.5	0.726	0.857
9	20	0.05	1.0	0.709	0.861
10	20	0.5	0.5	0.659	0.833
11	20	0.5	1.0	0.658	0.849
12	20	1.0	1.0	0.636	0.828
13	200	0.05	0.05	0.401	0.734
14	200	0.05	0.5	0.555	0.815
15	200	0.05	1.0	0.567	0.836
16	200	0.5	0.5	0.529	0.814
17	200	0.5	1.0	0.553	0.829
18	200	1.0	1.0	0.529	0.814
GCAE	-	-	-	0.757	0.928
popvae	-	-	-	0.788	0.939
t-SNE	-	-	-	0.808	0.906
UMAP	-	-	-	0.912	0.873
PCA	-	-	-	0.657	0.899

Table 10: UMAP models with different hyperparameter combinations, and their F1 scores for two classification models based on reduction to 2 dimensions. The last five rows show the same F1 score metrics for 2 dimensions for the models presented in the main article text.

References

[1] Leland McInnes, John Healy, and James Melville. *UMAP: Uniform Manifold Approximation and Projection for Dimension Reduction.* 2020. arXiv: 1802.03426 [stat.ML].

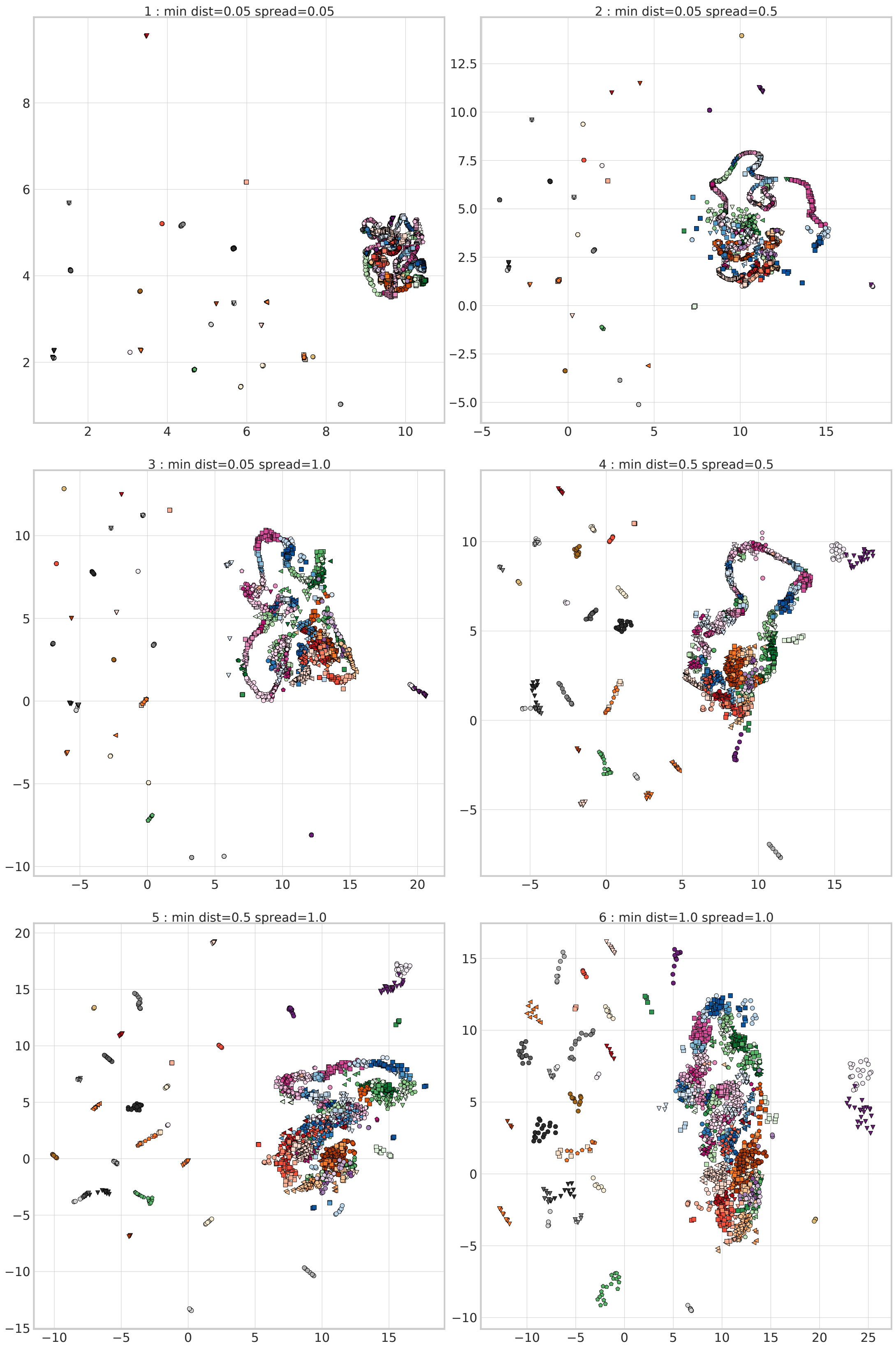

Figure 2: Dimensionality reduction results of UMAP models with number of neighbors = 3.

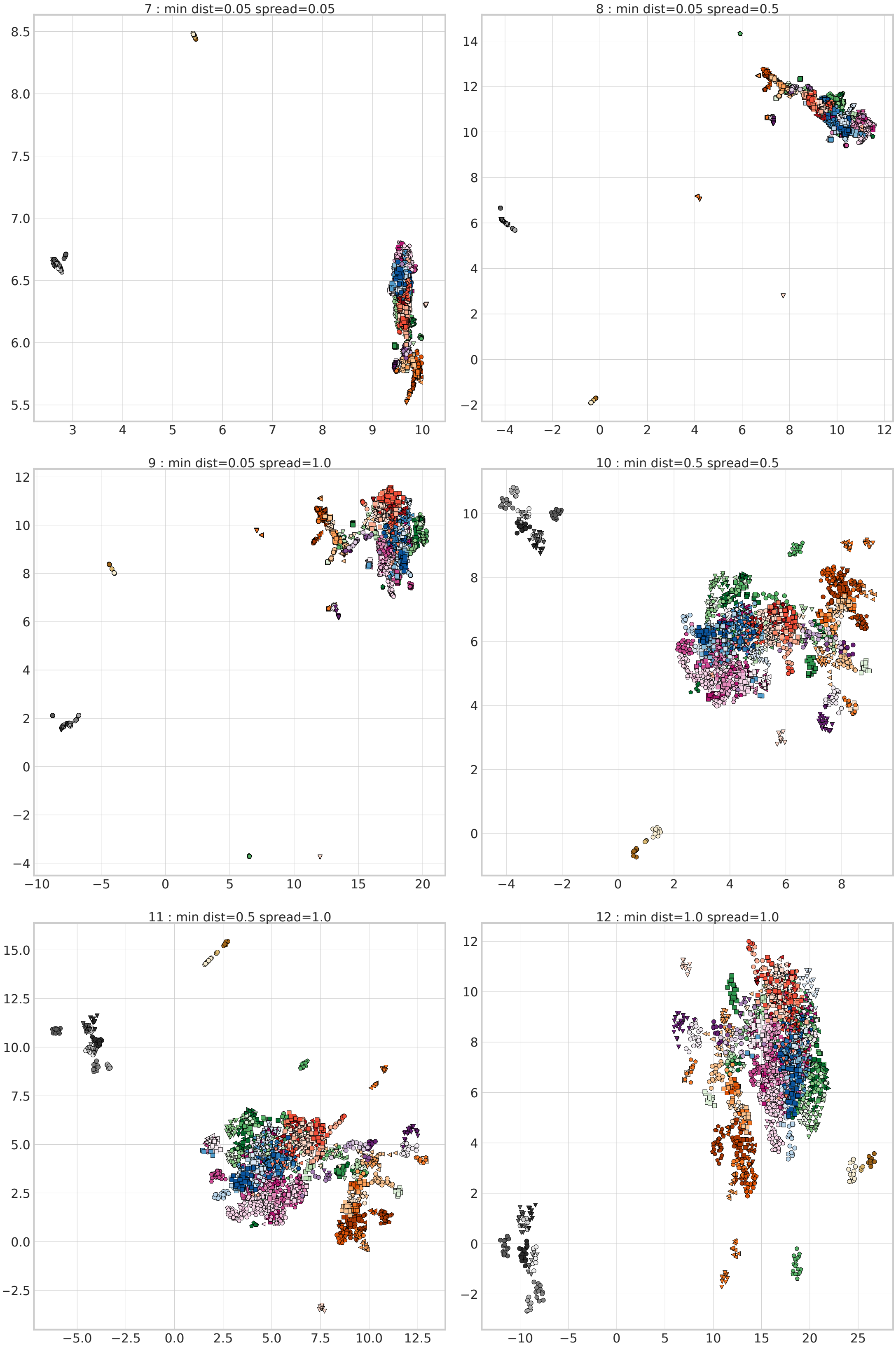

Figure 3: Dimensionality reduction results of UMAP models with number of neighbors = 20.

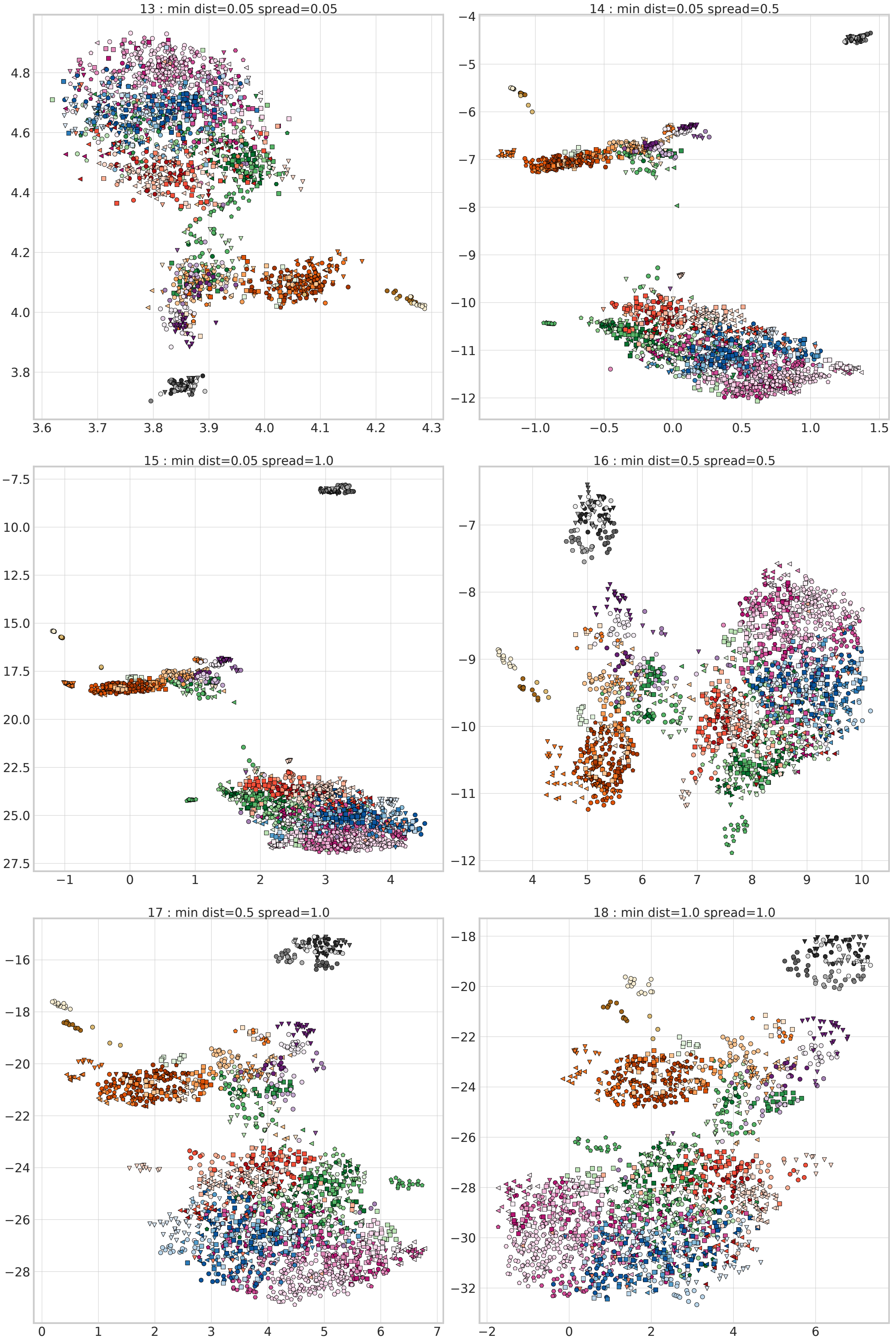

Figure 4: Dimensionality reduction results of UMAP models with number of neighbors = 200.
